# Ancient viral DNA in the human genome linked to neurodegenerative diseases

**DOI:** 10.1016/j.bbi.2024.10.020

**Published:** 2025-01

**Authors:** Rodrigo R.R. Duarte, Douglas F. Nixon, Timothy R. Powell

**Affiliations:** a Social, Genetic & Developmental Psychiatry Centre, Institute of Psychiatry, Psychology & Neuroscience, King’s College London, London, United Kingdom; b Division of Infectious Diseases, Weill Cornell Medicine, Cornell University, New York, NY, the United States of America; c Feinstein Institutes for Medical Research, Northwell Health, Manhasset, NY, the United States of America

**Keywords:** Retrotranscriptome-wide association studies, Statistical genetics, Neurodegeneration, Genome-wide association studies

## Abstract

**Background:**

Human endogenous retroviruses (HERVs) are sequences in the human genome that originated from infections with ancient retroviruses during our evolution. Previous studies have linked HERVs to neurodegenerative diseases, but defining their role in aetiology has been challenging. Here, we used a retrotranscriptome-wide association study (rTWAS) approach to assess the relationships between genetic risk for neurodegenerative diseases and HERV expression in the brain, calculated with genomic precision.

**Methods:**

We analysed genetic association statistics pertaining to Alzheimer’s disease, amyotrophic lateral sclerosis, multiple sclerosis, and Parkinson’s disease, using HERV expression models calculated from 792 cortical samples. Robust risk factors were considered those that survived multiple testing correction in the primary analysis, which were also significant in conditional and joint analyses, and that had a posterior inclusion probability above 0.5 in fine-mapping analyses.

**Results:**

The primary analysis identified 12 HERV expression signatures associated with neurodegenerative disease susceptibility. We found one HERV expression signature robustly associated with amyotrophic lateral sclerosis on chromosome 12q14 (MER61_12q14.2) and one robustly associated with multiple sclerosis on chromosome 1p36 (ERVLE_1p36.32a). A co-expression analysis suggested that these HERVs are involved in homophilic cell adhesion via plasma membrane adhesion molecules.

**Conclusions:**

We found HERV expression profiles robustly associated with amyotrophic lateral sclerosis and multiple sclerosis susceptibility, highlighting novel risk mechanisms underlying neurodegenerative disease, and offering potential new targets for therapeutic intervention.

## Highlights

• Retrotranscriptome-wide association studies identify HERV expression signatures associated with neurodegenerative diseases.
• MER61_12q14.2 and ERVLE_1p36.32a are robustly linked to risk for amyotrophic lateral sclerosis and multiple sclerosis.
• These HERVs may play a role in homophilic cell adhesion via plasma membrane adhesion molecules.

## 1 Introduction

Human endogenous retroviruses (HERVs) are sequences in our genome that originated from infections with ancient retroviruses during our evolution (Göke and Ng, 2016). These sequences comprise approximately 8 % of our genome, and most are considered regulatory. However, there are 14,968 sequences annotated as putative HERV transcriptional units, comprising of remnants of viral genes such as polymerase (*pol*), envelope (*env*)*,* and antigen (*gag*) flanked by former viral promoters, which may produce non-coding RNAs and proteins (Bendall et al., 2019). HERVs have been hypothesised to activate innate immunity through DNA sensing via the cyclic GMP–AMP synthase (cGAS)–stimulator of interferon genes (STING) pathway. They have also been suggested to produce proteins that can be targeted by adaptive immunity, potentially leading to recognition and attack by the immune system (Humer et al., 2006). Additionally, they have been implicated in processes such as the formation of the placenta, determining cell fate, and mediating our body’s response to viral infections (Dopkins and Nixon, 2024, Marston et al., 2021).

Previous research has identified abnormal HERV expression profiles in neurodegenerative diseases such as Alzheimer’s disease (Dembny et al., 2020), amyotrophic lateral sclerosis, also known as motor neurone disease (Li et al., 2015), multiple sclerosis (Perron et al., 2012), and Parkinson’s disease (Perron et al., 2012). However, most studies have been unable to distinguish expression mechanisms involved in disease aetiology from those that co-occur or result from downstream disease pathology or treatment. Retrotranscriptome-wide association studies (rTWAS), which pinpoint expression signatures tied to genetic variants associated with disease risk, provide a powerful means to infer HERVs involved in disease aetiology (Duarte et al., 2024b). This framework uses results from genome-wide association studies (GWAS) encompassing hundreds of thousands of individuals, which compare the frequency of genetic variants in cases and controls. These data can then be cross-referenced with an expression quantitative trait loci (eQTL) database, to infer the effect genetic risk exerts on the expression of HERVs competitively against canonical protein coding genes, in a tissue of interest.

In the present study, we employed a multi-step retrotranscriptome-wide association study approach to identify HERVs involved in the aetiology of Alzheimer’s disease, amyotrophic lateral sclerosis, multiple sclerosis, and Parkinson’s disease. We investigated how genetic risk factors for these neurodegenerative diseases impacted cortical HERV expression. Our findings revealed two HERVs that are downregulated in association with disease risk, including one on chromosome 12q14 linked to amyotrophic lateral sclerosis, and another on chromosome 1p36 associated with multiple sclerosis. Ultimately, we have identified novel, robust HERV risk mechanisms for neurodegenerative diseases.

## 2 Materials & methods

### 2.1 GWAS summary statistics processing

We obtained summary statistics from the largest to-date GWAS of Alzheimer’s disease (de Rojas et al., 2021), amyotrophic lateral sclerosis (van Rheenen et al., 2021), multiple sclerosis (International Multiple Sclerosis Genetics Consortium, 2019), and Parkinson’s disease (Kim et al., 2024). These studies analysed mainly individuals of European ancestry, although the amyotrophic lateral sclerosis study also included a smaller proportion of individuals of East Asian ancestry, and the Parkinson’s disease study also comprised a smaller proportion of individuals of East Asian, Latin American, and African ancestry. We also analysed results of a GWAS of Parkinson’s disease performed in individuals of African ancestry (Rizig et al., 2023). No other relevant large studies were identified in the GWAS Catalog. We analysed only biallelic non-ambiguous single nucleotide polymorphisms (SNPs) with imputed minor allele frequency > 5 % (calculated based on the European or African subset of the 1,000 Genomes reference panel), and imputation score > 0.80, when this information was available.

### 2.2 Retrotranscriptome-wide association studies (rTWAS)

The rTWAS were performed through analysis of GWAS summary statistics using FUSION (Gusev et al., 2016) and our customised SNP weight panels (Duarte et al., 2024b). The weights correspond to expression quantitative loci (eQTL) databases that inform the expression of canonical genes and HERVs in the dorsolateral prefrontal cortex, according to genotype within 1 Mb *cis* windows. The weights were previously constructed using the European (N = 563) or African American (N = 229) subsets of the CommonMind Consortium. The CommonMind Consortium dataset is a large-scale genomic resource that includes gene expression and genotyping data from autopsy human brain tissue, collected from four brain banks and processed across independent phases (Fromer et al., 2016, Hoffman et al., 2019). It comprises individuals diagnosed with schizophrenia (38.3 %), bipolar disorder (12.6 %), a general affective disorder (1.0 %), as well as unaffected controls (48.1 %), while excluding those with evidence of a neurodegenerative disease diagnosis (Hoffman et al., 2019). A comprehensive description of how the data was processed and how we corrected for confounders such as psychiatric diagnosis, can be found elsewhere (Duarte et al., 2024b). Since the largest genetic studies identified for our analysis predominantly involved individuals of European ancestry, we performed the rTWAS using the European weights and the corresponding subset of the CommonMind Consortium as the reference population to control for linkage disequilibrium. For the only non-European neurodegenerative disease GWAS with matching SNP weights—Parkinson’s disease in Africans—we used the African American weights and the corresponding subset of the CommonMind Consortium as the reference population. We applied multiple testing correction to the rTWAS association signals per trait using the Bonferroni method, considering the total number of tested genetic features (P value cut-off = 6.10 × 10^−6^ for signatures identified using the European weights and 7.92 × 10^−6^ for those identified using the African American weights). Plots were generated using the FUSION pipeline and scripts adapted from https://github.com/rodrigoduarte88/hiv-*meta*-twas-2021 (Duarte et al., 2022) and https://github.com/rodrigoduarte88/TWAS_HERVs-SCZ (Duarte et al., 2024b).

### 2.3 rTWAS secondary analyses

We performed sensitivity analyses to test whether HERV expression signals were able to explain GWAS signals competitively against canonical genes. To achieve this, we performed conditional analyses using FUSION (Gusev et al., 2016) to estimate the proportion of the GWAS signals that were explained by rTWAS signals within each locus. We also performed fine-mapping analyses using FOCUS (Mancuso et al., 2019) to identify the strongest expression association signal within each linkage disequilibrium block after controlling for the correlation of neighbouring signals, for GWAS variants that surpassed genome-wide significance (P < 5 x 10^-8^). FOCUS calculates the posterior inclusion probability (PIP) for each expression signature in an LD block to be causal given the observed rTWAS statistics, whereby those with PIP > 0.50 are more likely to be causal than other features at the locus.

### 2.4 Statistical analyses

Analyses were performed using King’s College London’s High Performance Computing Cluster CREATE (King's College London, 2024), in Bash 5.0.17 (GNU Project Bourne Again SHell) and R 3.6.3 (The R Project for Statistical Computing, Vienna, Austria).

## 3 Results

### 3.1 Retrotranscriptome-wide association studies (rTWAS)

**Fig. 1:**
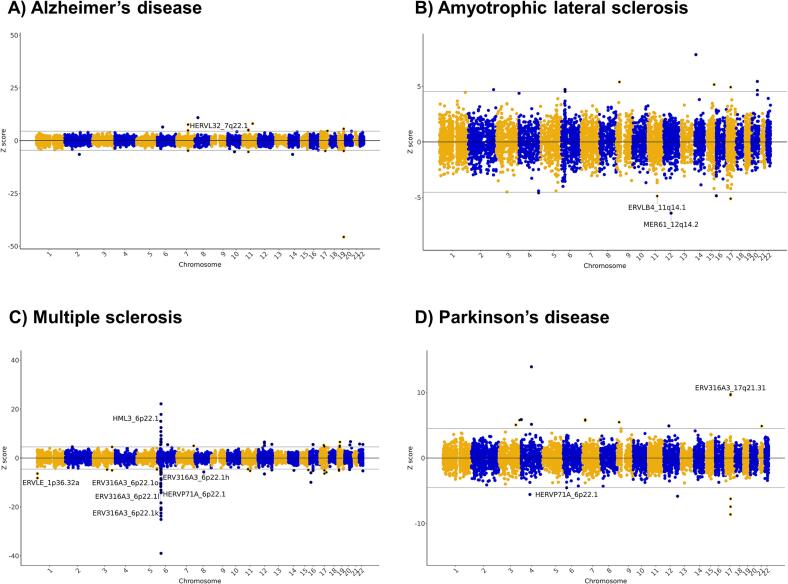
**Retrotranscriptome-wide association studies of neurodegenerative diseases.** Manhattan biplots showing expression signatures significantly associated with **(A)** Alzheimer’s disease, **(B)** amyotrophic lateral sclerosis, **(C)** multiple sclerosis, and **(D)** Parkinson’s disease. These studies analysed cohorts primarily of European ancestry. Results from the rTWAS of Parkinson’s disease in Africans is not shown, as it did not identify significant HERV expression signatures associated with risk. In these graphs, the X-axis indicates genomic location and the Y-axis shows the rTWAS Z score. The horizontal grey lines indicate transcriptome-wide significance, i.e., a threshold adjusted for the number of expressed features using the Bonferroni method (P value cut-off = 6.10 × 10^−6^). Only Bonferroni-significant HERV features are labelled.

Analysis of the primarily European GWAS using the rTWAS approach identified 12 HERV expression signatures in total associated with neurodegenerative diseases after correcting for multiple testing using the Bonferroni method (P value cut-off = 6.10 × 10^−6^, Supplemental Table 1**,** Fig. 1). More specifically, for Alzheimer’s disease, we identified 17 expression signatures associated with genetic risk, but only one was a HERV (HERVL32_7q22.1, Z = 4.9, P = 1.14 x 10^-6^). For amyotrophic lateral sclerosis, we identified 14 expression signatures associated with risk, of which two pertained to HERVs (MER61_12q14.2, Z = −6.4, P = 1.5 x 10^-10^; ERVLB4_11q14.1, Z = −4.9, P = 1.12 x 10^-6^). For multiple sclerosis, we identified 80 expression signatures associated with risk, of which seven were HERVs. These included six HERVs located at the major histocompatibility complex (MHC) locus, and one located elsewhere (ERVLE_1p36.32a, Z = −8.1, P = 2.81 x 10^-16^). For Parkinson’s disease, we identified 18 expression signatures associated with genetic predisposition, of which two were HERVs, including one located at the MHC locus and another on chromosome 17q21 (ERV316A3_17q21.31, Z = 9.7, P = 2.06 x 10^-22^). Analysis of the African GWAS of Parkinson’s disease identified one expression signature associated with genetic risk (P value cut-off = 7.92 × 10^−6^), but this did not correspond to a HERV. While these results provide evidence of association between specific HERVs and these neurodegenerative diseases, additional analyses are required to ensure that they are likely to represent processes involved in aetiology. This is particularly important for signatures originating from the MHC locus, which are challenging to interpret due to the complex linkage disequilibrium structure in this region.

### 3.2 Conditional analyses further support the role of HERVs in neurodegenerative diseases

**Fig. 2:**
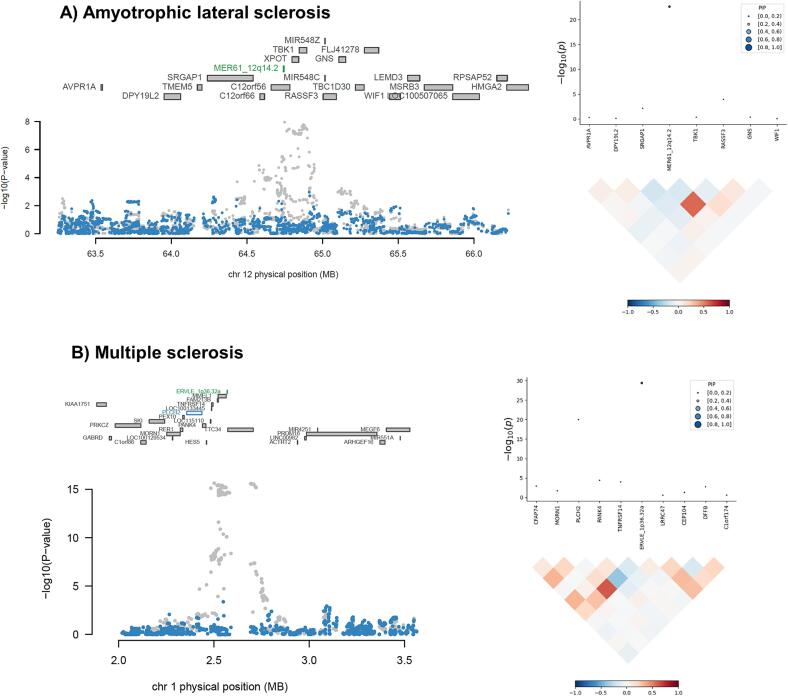
**Conditional and joint analyses, and fine-mapping results.** Sensitivity analyses support a role for **(A)** MER61_12q14.2 in amyotrophic lateral sclerosis and **(B)** ERVLE_1p36.32a in multiple sclerosis. On the left, panels show results of the conditional and joint analyses: the top part indicates the genomic context, whereas the lower part contains a graph in which the X-axis indicates genomic location, and the Y-axis shows −log10(P) of genetic variant associations from the GWAS, before (grey dots) and after (blue dots) conditioning on jointly significant genes in each locus. On the right, panels show results of the fine-mapping analyses: the lower part displays the correlation of predicted expression across genetic features at the locus, whereas the top part contains a graph where the Y-axis indicates the rTWAS association P value (two-sided), and the X-axis shows genetic features in the linkage disequilibrium block. The size and colour of the points indicate the posterior inclusion probability (PIP), indicating the probability that the expression feature is causal for the association signal at the locus. (For interpretation of the references to colour in this figure legend, the reader is referred to the web version of this article.)

We performed conditional and joint analyses within FUSION (Gusev et al., 2016) to isolate HERV expression associations that are independent from the expression of surrounding canonical genes and that are more likely to explain the GWAS signal in their loci (Supplemental Table 2**,** Fig. 2). For Alzheimer’s disease, we identified 13 expression signals that were significant in the joint and conditional analyses, but none of these were HERVs. For amyotrophic lateral sclerosis, we identified 13 significant expression signals, of which two were HERVs (MER61_12q14.2, joint Z = −6.4, joint P = 1.5 x 10^-10^; ERVLB4_11q14.1, joint Z = −4.9, joint P = 1.1 x 10^-6^). For multiple sclerosis, we identified 55 significant expression signals, of which five were HERVs, including four from the MHC locus and one located on chromosome 1p36 (ERVLE_1p36.32a, joint Z = −8.2, joint P = 2.8 x 10^-16^). For Parkinson’s disease, we identified 12 significant expression signals, of which two were HERVs, including one from the MHC locus and one located on chromosome 17q21 (ERV316A3_17q21.31, joint Z = 9.7, joint P = 2.1 x 10^-22^). For Parkinson’s disease in the African study, we identified an expression signal that was significant in the joint and conditional analyses, but it was not a HERV.

### 3.3 HERV expression as aetiological risk mechanisms for neurodegenerative diseases

We have taken a conservative approach to infer HERV expression signatures involved in the aetiology of neurodegenerative diseases. The approach involved identifying expression signatures that survived multiple testing correction in the primary rTWAS using the Bonferroni method, that were also significant in conditional and joint analyses within FUSION (Gusev et al., 2016), and that had a posterior inclusion probability (PIP) above 0.5 in a fine-mapping analysis using FOCUS (Mancuso et al., 2019) (Supplemental Table 3). This multi-step approach identified one expression signature robustly associated with amyotrophic lateral sclerosis on chromosome 12q14 (MER61_12q14.2, rTWAS and joint Z = −6.4, rTWAS and joint P = 1.5 x 10^-10^, rTWAS Bonferroni P = 1.2 x 10^-6^, PIP = 1.0), and one associated with MS on chromosome 1p36 (ERVLE_1p36.32a, rTWAS and joint Z = −8.2, rTWAS and joint P = 2.8 x 10^-16^, rTWAS Bonferroni P = 2.3 x 10^-12^, PIP = 1.0). Using this approach, we also identified three expression signatures originating from the MHC locus in association with multiple sclerosis (ERV316A3_6p22.1, HERVP71A_6p22.1 and HML3_6p22.1; PIP = 1.0). However, considering the complex linkage disequilibrium structure of this locus, we interpret these results with caution, as they are still likely to correlate with risk attributed to other genetic elements in the location.

### 3.4 Validity of the multi-step approach

To demonstrate the validity of our multi-step approach, we also identified robust expression signatures relating to canonical genes previously identified in association with the conditions investigated. For instance, we identified a robust association between Alzheimer’s disease and *TOMM40* (rTWAS and joint Z = −45.6, rTWAS, joint P and rTWAS Bonferroni P < 1.0 x 10^-139^, PIP = 1.0). This gene is located at the *APOE* locus and has been hypothesised to play a role in disease susceptibility independently from *APOE*, via effects on mitochondrial function (Lee et al., 2021). Further, we replicated the association between amyotrophic lateral sclerosis and *C9orf72* (rTWAS and joint Z = 5.4, rTWAS and joint P = 6.8 x 10^-8^, rTWAS Bonferroni P = 5.49 x 10^-4^, PIP = 1.0), one of the most well-known genetic risk factors for this condition. We also observed a strong association between multiple sclerosis and *BACH2* (rTWAS Z and joint Z = 5.5, rTWAS P and joint P = 4.8 x 10^-8^, rTWAS Bonferroni P = 4.0 x 10^-4^, PIP = 1.0), a gene encoding a transcription factor that regulates T helper (Th) 2 immune response, which moderates adaptive immune response against various allergens and infections, and has been implicated in this condition (Liu and Liu, 2022). We also identified a robust association between Parkinson’s disease and *SNCA* (rTWAS Z and joint Z = 13.9, rTWAS P and joint P = 3.8 x 10^-44^, rTWAS Bonferroni P = 3.1 x 10^-40^, PIP = 1.0), which encodes alpha-synuclein, the protein that makes up Lewy bodies, i.e., aggregates of protein that develop inside nerve cells that appear to contribute to neurodegeneration and are often seen in brain autopsies of individuals with Parkinson’s disease (Chen et al., 2020).

### 3.5 Inferring the function of high confidence risk HERVs

There is currently a gap in the literature pertaining to the function of locus specific HERVs. In Duarte et al. (2024b), our group analysed expression data from 563 dorsolateral prefrontal cortex samples of European individuals using weighted correlation network analysis (WGCNA) (Langfelder and Horvath, 2008), to infer the function of expressed HERVs. This analysis was performed based on the premise that genetic features that are expressed together are more likely to share a similar function. In that study, we found that MER61_12q14.2 and ERVLE_1p36.32a belonged to the ‘magenta’ module (see Extended Data 4 and 5 from Duarte et al. (2024b)). This module was significantly associated with the gene ontology (GO) term “*GO:0007156 homophilic cell adhesion via plasma membrane adhesion molecules*” (P = 2.3 x 10^-9^, Bonferroni P = 8.4 x 10^-4^, number of genes in GO term = 430, number of genes in module = 151, number of overlapping genes = 21, enrichment ratio = 4.8). Interestingly, and in contrast to our findings here focused on neurodegenerative diseases, the HERVs identified in association with schizophrenia, bipolar disorder, and depression genetics in Duarte et al. (2024b) belonged to a module enriched for GO terms linked to signal transduction.

## 4 Discussion

In our study, we interrogated large-scale genetic studies of neurodegenerative diseases using customised eQTL databases that enabled us to infer HERV expression profiles associated with neurodegeneration. We identified 12 HERV expression signatures associated with neurodegenerative diseases, including one linked to Alzheimer’s disease, two to amyotrophic lateral sclerosis, seven to multiple sclerosis, and two to Parkinson’s disease. To identify HERV expression signatures more likely to be involved in disease aetiology, we further employed a multi-step approach using conditional and joint analyses, as well as fine-mapping. This led to the identification of one robust non-MHC expression signature each for amyotrophic lateral sclerosis and multiple sclerosis.

One key strength of our study relative to previous research on HERV expression in neurodegenerative diseases is our focus on the effects of disease-associated genetic variants on HERV regulation. Prior case-control studies often captured expression changes driven by disease pathology or treatment (Gusev et al., 2016). In our study, by harnessing eQTL data, we were able to isolate the impact of specific genetic loci on HERV expression, whilst minimising confounding factors such as the effect of disease progression or treatment on expression profiles.

Also differently from previous research, our approach considers HERV transcriptional units as independent genetic features. Most HERV research to date has been conducted using quantification approaches such as RT-qPCR, Western blotting or microarrays, which group expression signals arising from many distinct locations across the genome due to their sequence similarity. This past research, coupled with functional genetic approaches like overexpression of consensus HERV sequences in model organisms, has provided compelling evidence linking HERV expression with neurodegenerative diseases, particularly multiple sclerosis (Duperray et al., 2015) and amyotrophic lateral sclerosis (Li et al., 2015). Notably, research into multiple sclerosis in the 1990s led to the discovery of the Multiple Sclerosis-Associated Retrovirus (MSRV), a member of the HERV-W family (Perron et al., 1997). Although the lack of precision in assessing HERV expression has previously limited our understanding of their role in disease, there have been two clinical studies that aimed to target HERV expression in amyotrophic lateral sclerosis (Adler et al., 2024), and four in relation to multiple sclerosis (Diebold and Derfuss, 2019). This highlights the growing interest in therapeutic strategies that target HERVs as potential modulators of neurodegenerative disease progression.

HERV expression has been thought to play a role in neurodegeneration by modulating various aspects of neuroinflammation, including by activating innate immunity through DNA sensing (Dopkins and Nixon, 2024), by regulating immune responses through interaction with pathogens such as Epstein-Barr virus (EBV) (Meier et al., 2021) or by regulating Toll-like receptors (TLRs) (Duperray et al., 2015). In our study, we identified specific HERVs expressed in the cortex that are associated with amyotrophic lateral sclerosis and multiple sclerosis. We found evidence that suggests that these HERVs may be involved in homophilic cell adhesion via plasma membrane adhesion molecules, a process previously linked to neurodegeneration (Adewuyi et al., 2022, Zhou et al., 2023). Disruption of homophilic cell adhesion could contribute to neurodegeneration by impairing the maintenance of neural networks, synaptic stability, or myelination, ultimately leading to cell death. Despite these findings, the precise role of the HERVs we identify here remains unclear, and future functional studies are essential to elucidate the molecular and cellular mechanisms underlying their involvement in these conditions.

Our study provides an important step forward in our understanding of how ancient viral DNA in the human genome contributes to neurodegenerative disease, but some limitations should be acknowledged. First, we assessed how genetic risk for neurodegenerative diseases impacts HERV expression, but it remains unclear how environmental risk factors may affect HERV expression in relation to disease risk. This is relevant since infections are known to cause changes to HERV expression (Marston et al., 2021, Uleri et al., 2014). Second, we investigated the impact of genetic risk only on cortical HERV expression, but it is plausible that there are regulatory mechanisms linked to susceptibility in additional brain areas and tissues, including in blood. Further studies analysing how genetic risk imparts its effects on HERV expression across relevant tissues are warranted. Third, our study analysed the largest genetic association studies of neurodegenerative diseases published to date, which consisted primarily of individuals of European ancestry. We applied our approach to a GWAS of Parkinson’s disease in Africans, but the analysis was constrained by the small power of that study, and we remain challenged by the limited availability of genetic studies from non-European cohorts. Future studies investigating genetic and expression differences in more diverse cohorts are likely to highlight shared and ancestry-specific mechanisms.

## CRediT authorship contribution statement

**Rodrigo R.R. Duarte:** Writing – review & editing, Writing – original draft, Software, Methodology, Investigation, Funding acquisition, Formal analysis, Data curation, Conceptualization. **Douglas F. Nixon:** Writing – review & editing, Funding acquisition, Conceptualization. **Timothy R. Powell:** Writing – review & editing, Funding acquisition, Conceptualization.

## Funding

Research reported in this publication was supported by the National Institutes of Health (NIH) under award number R21 HG011513 to T.R.P., D.F.N. and R.R.R.D., and a Psychiatry Research Trust grant to T.R.P. and R.R.R.D. The content is solely the responsibility of the authors and does not necessarily represent the official views of the NIH. T.R.P. is supported by an MRC (UKRI) New Investigator Research Grant (MR/W028018/1). For the purpose of open access, the author has applied a CC BY public copyright license to any Author Accepted Manuscript version arising from this submission. This research is also part-funded by the National Institute for Health and Care Research (NIHR) Maudsley Biomedical Research Centre at South London and Maudsley National Health Service (NHS) Foundation Trust and King’s College London. The views expressed are those of the authors and not necessarily those of the NHS, the NIHR or the Department of Health and Social Care.

## Declaration of competing interest

The authors declare that they have no known competing financial interests or personal relationships that could have appeared to influence the work reported in this paper.

## Data Availability

We obtained GWAS summary statistics of Alzheimer’s disease (de Rojas et al., 2021), amyotrophic lateral sclerosis (van Rheenen et al., 2021), multiple sclerosis (International Multiple Sclerosis Genetics Consortium, 2019), and Parkinson’s disease (Kim et al., 2024, Rizig, et al., 2023) by accessing the online repositories provided by the authors. SNP weights and population reference panels for rTWAS can be downloaded from the King’s College London Research Data Repository (KORDS) (https://doi.org/10.18742/22179655) (Duarte, et al., 2024c). All code used in the manuscript is available from GitHub (https://github.com/rodrigoduarte88/rTWAS_neurodegen) and the King’s College London Research Data Repository (KORDS) (https://doi.org/10.18742/26117281) (Duarte, et al., 2024a, Duarte et al., 2024b).
